# Ischemic stroke risk factors not included in the CHADS-VASC score in patients with non-valvular atrial fibrillation

**DOI:** 10.1055/s-0043-1771167

**Published:** 2023-08-11

**Authors:** Laurine Le Goff, Stanislas Demuth, Andreas Fickl, Lucian Muresan

**Affiliations:** 1 “Emile Muller” Hospital, Department of Cardiology, Mulhouse, France.; 2 Strasbourg University Hospital, Department of Neurology, France.; 3 “Emile Muller” Hospital, Department of Neurology, Mulhouse, France.

**Keywords:** Atrial Fibrillation, Stroke, Risk Factors, CHA _2_ DS _2_ -VASc, Fibrilação Atrial, Acidente Vascular Cerebral, Fatores de Risco, CHA2DS2-VASc

## Abstract

**Background** In patients with atrial fibrillation, the CHA _2_ DS _2_ -VAS _C_ score guides stroke prevention using anticoagulants, but it is an imperfect score. Other potential risk factors such as renal failure, the type of atrial fibrillation, active smoking, cancer, sleep apnea or systemic inflammation have less well been investigated.

**Objective** To assess the impact of these factors on ischemic stroke risk in patients with non-valvular atrial fibrillation.

**Methods** On a population of 248 patients (124 patients with acute ischemic stroke and 124 controls), we performed a logistic regression to assess the impact of multiple non-classic risk factors for the prediction of acute ischemic stroke. Their impact on mortality was assessed by performing a survival analysis.

**Results** A high CHA _2_ DS _2_ -VASc score (OR 1.75; 95% CI 1.13–2.70; p = 0.032), treatment with anticoagulants (OR 0.19; 95% CI 0.07–0.51; p < 0.001) and permanent atrial fibrillation (OR 6.31; 95% CI 2.46–16.19; p < 0.001) were independently associated with acute ischemic stroke. Renal failure and chronic obstructive pulmonary disease predicted a higher mortality. After adjusting for age, sex, the CHA _2_ DS _2_ -VASc score and the use of anticoagulants, the only risk factor predictive for acute ischemic stroke was the permanent type of AF (OR: 8.0 [95% CI 2.5–25.5], p < 0.001).

**Conclusions** The CHA _2_ DS _2_ -VASc score, the absence of anticoagulants and the permanent type of atrial fibrillation were the main predictive factors for the occurrence of acute ischemic stroke. Larger studies are necessary for conclusive results about other factors.

## Resumo

**Antecedentes** Em pacientes com fibrilação atrial, o escore CHA2DS2-VASC orienta a prevenção de AVC com anticoagulantes, mas é um escore imperfeito. Outros fatores de risco potenciais, como insuficiência renal, o tipo de fibrilação atrial, tabagismo ativo, câncer, apnéia do sono ou inflamação sistêmica foram menos bem investigados.

**Objetivo** Avaliar o impacto desses fatores no risco de AVC isquêmico em pacientes com fibrilação atrial não valvular.

**Métodos** Em uma população de 248 pacientes (124 pacientes com AVC isquêmico agudo e 124 controles), realizamos uma regressão logística para avaliar o impacto de múltiplos fatores de risco não clássicos na predição de AVC isquêmico agudo. O seu impacto na mortalidade foi avaliado através da realização de uma análise de sobrevivência.

**Resultados** Escore CHA2DS2-VASc alto (OR 1,75; IC 95% 1,13–2,70; p = 0,032), tratamento com anticoagulantes (OR 0,19; IC 95% 0,07–0,51; p < 0,001) e fibrilação atrial permanente (OR 6,31; 95% CI 2,46–16,19; p < 0,001) foram independentemente associados ao AVC isquêmico agudo. Insuficiência renal e doença pulmonar obstrutiva crônica previram maior mortalidade. Após ajuste para idade, sexo, pontuação CHA2DS2-VASc e uso de anticoagulantes, o único fator de risco preditivo para AVC isquêmico agudo foi o tipo permanente de FA (OR: 8,0 [IC 95% 2,5–25,5], p < 0,001).

**Conclusões** O escore CHA2DS2-VASc, a ausência de anticoagulantes e o tipo permanente de fibrilação atrial foram os principais fatores preditivos para a ocorrência de AVC isquêmico agudo. Estudos maiores são necessários para resultados conclusivos sobre outros fatores.

## INTRODUCTION

On a global scale, stroke represents 16.9 million cases each year according to the “Global burden of disease 2010” study. In Europe, 80% are ischemic and 19% to 37% of them are of cardio-embolic origin. 1 In 2009, the French PMSI registry recorded 106 927 strokes. 2 Stroke is the leading cause of adult motor handicap in France. 3 With 143 000 cases recorded in 2015, stroke is a public health concern, with a mean hospital cost of 34 638 euros per patient, five years following the event. 4

Atrial fibrillation (AF) affects 1.5 to 2% of the general population, 5 representing 33 million people worldwide. 6 Its incidence increases with age and it multiplies by 5 the global risk for acute ischemic strokes (AIS) among elderly. 7 The estimated annual risk of AIS for an AF patient is 5-7%. 8

To prevent cardio-embolic AIS among patients with non-valvular atrial fibrillation (NV-AF), several scores have been proposed over the years, but lack exhaustivity: the score AFI, 9 SPAF, 10 CHA _2_ DS _2_ , 11 CHA _2_ DS _2_ -VASc, 12 ATRIA, 13 14 ABC, 15 GARFIELD-AF, 16 R _2_ CHADS _2_ , 17 CHA _2_ DS _2_ -VASc-RAD = F. 18

The European Society of Cardiology maintains the CHA _2_ DS _2_ -VASc score as the gold standard in its latest guidelines in 2016. 19 Nonetheless, this score remains improvable as more recent scores with a wider range of variables have better predictive values. Indeed, recent studies suggest other risk factors: renal failure, the type of atrial fibrillation, sleep apnea, systemic inflammation, cancer, smoking, ethnicity, chronic obstructive pulmonary disease (COPD), obesity, genetics and alcohol abuse. 13 20 21

In this study, we investigate the relationship between some of these additional factors and the occurrence of cardio-embolic AIS among NV-AF patients.

## METHODS

To conduct this case-control study, we examined the electronic health record from the neurovascular unit (cases) and from the department of cardiology (controls) of the medical center of Mulhouse, France. All patients included were hospitalized in one of these two departments.

As cases, we included patients with NV-AF presenting to the neurovascular unit for cardio-embolic AIS from 01.01.2018 to 30.09.2019 and aged over 18. As controls, we included patients presenting to the department of cardiology from 1/1/2018 to 12/31/2019 with a NV-AF without history of AIS and aged over 18. We excluded patients with valvular atrial fibrillation or ancient history of stroke. Cases and controls are included in chronological order and matched on sex.

This study was approved by the ethical committee of the Mulhouse Medical Center.

Paroxysmal atrial fibrillation was defined as atrial fibrillation that lasted less than 7 days, or that required cardioversion for termination after more than 7 days. Persistent AF was defined as AF lasting for more than 7 days, or that required cardioversion for termination after more than 7 days. Permanent AF was considered AF that lasted for more than 1 year and that was accepted both by the patient and the clinician, and no strategy of rhythm control was applied to try to obtain sinus rhythm.

Statistical analysis was performed using the Statistical Package for the Social Sciences (SPSS Inc. Chicago, Illinois) version 22. Descriptive statistics were used to summarize patients' characteristics. Normality was assessed for all continuous variables using the Shapiro_Wilk test. When the assumption held, results were expressed as mean ± standard deviation (SD) or otherwise by median ± interquartile range. Categorical variables were presented as counts and proportions (%).

The Chi square test was used to compare different categorical characteristics of the patients and associated diseases. According to the sample size of the compared patient populations, the t test for independent samples / paired samples or Mann-Whitney U test were used to compare the age, FEVG, CHA _2_ DS _2_ -VASc and HAS-BLED score, levels of CRP, renal function parameters, apnea – hypopnea index for patients with obstructive sleep apnea syndrome from both groups.

Pearson or Spearman's correlation coefficients were used to test the association between several potential risk factors and the presence of stroke.

We drew receiver operating characteristic (ROC) curves and calculated the area under the curve AUC) to compare the sensitivity and specificity of several parameters in predicting the presence of stroke in the present patient population.

To determine predictors of stroke, univariate and multivariate logistic regression were performed. Multivariate logistic regression model included all variables found to be predictors of stroke in univariate analyses, maintaining an adequate event per predictor variable value. Variables included in these models were both qualitative and quantitative variables.

A p value of < 0.05 was considered statistically significant.

## RESULTS

In total, we included 248 patients: 124 cases of AIS (59 males and 65 females) and 124 controls (59 males and 65 females). The mean age among cases was 77.8 years vs. 69.7 years in the control group, a difference which was statistically significant (p < 0.01). We noticed a high prevalence of hypertension and dyslipidemia in both groups ( Table 1 ). Of all 124 patients in the case group, 34.7% had a personal history of atrial fibrillation.

**Table 1:** General characteristics of the patients

Characteristic		AIS group	Control group	Total
Number of patients n (%)		124 (50)	124 (50)	248 (100)
Sex, males n (%)		59 (47.5)	59 (47.5)	118 (47.5)
Mean age (years)		77.8 ± 10.6	69.7 ± 10.3	73.79 ± 11.25**
Height(m)		1.67 ± 0.10	1.70 ± 0.10	1.68 ± 0.10**
Weight (kg)		75.4 ± 17.1	83.9 ± 23.2	79.7 ± 20.8**
BMI (kg/m ^2^ )		27.4 ± 5.8	29.1 ± 7.2	28.3 ± 6.6*
• Cardiovascular risk factors n (%)	• High blood pressure	102 (82.25)	73 (58.87)	175 (70.56)**
	• Diabetes	31 (25)	28 (22.5)	59 (23.8)
	• Active smoking	14 (11.3)	14 (11.3)	28 (11.3)
	• Past smoking	28 (22.6)	32 (25.8)	60 (24.2)
	• Dyslipidemia	63 (50.8)	49 (39.5)	112 (45.1)*
	• Coronary artery disease	5 (4)	18 (14.5)	23 (9.2)**
• Comorbidities n (%)	• Asthma	5 (4)	2 (1.6)	7 (2.8)
	• COPD	4 (3.2)	11 (8.8)	15 (6)
	• Peripheral artery disease	9 (7.2)	3 (2.4)	12 (4.8)
	• Dilatation of the ascending aorta	2 (1.6)	6 (4.8)	8 (3.2)
	• Hyperthyroidism	4 (3.2)	10 (8)	14 (5.6)
	• Hypothyroidism	17 (13.7)	12 (9.6)	29 (11.7)
	• Osteoporosis	1 (0.8)	3 (2.4)	4 (1.6)
	• Alcoholism	6 (4.8)	5 (4)	11 (4.4)
• Initial type of atrial fibrillation: n (%)	• Paroxysmal	57 (45.9)	48 (38.7)	105 (42.3)
	• Persistent	35 (28.2)	61 (49.2)	96 (38.7)
	• Permanent	32 (25.8)	15 (12)	47 (18.9)
Mean CHA _2_ DS _2_ -VASc score		3.6 ± 1.4	2.8 ± 1.5	3.2 ± 1.5**
Mean HAS-BLED score		1.9 ± 0.8	1.5 ± 1.2	1.7 ± 1.0**
Current anticoagulant treatment n (%)		38 (30.6)	117 (94.3)	155 (62.5)**
• LVEF (Ultrasonography)	• Mean (%)	55.7 ± 11.6	52.9 ± 12.6	54.1 ± 12.2
	• ≥ 55%	68 (54.8)	72 (58)	140 (56.4)
	• 45–54%	3 (2.4)	10 (8)	13 (5.2)
	• 35–44%	10 (8)	17 (13.7)	27 (10.9)
	• < 35%	6 (4.8)	14 (11.2)	20 (8)
	• Not available	37 (29.8)	11 (8.8)	48 (19.3)
• Renal function	• GFR at entry, ml/min/1.73 m ^2^	64 ± 20	71 ± 22	67 ± 35*
	• GFR at discharge, ml/min/1.73 m ^2^	64 ± 21	70 ± 22	65 ± 35
	• Creatinine at entry, mmol/l	94 ± 39	90 ± 38	92 ± 35
	• Creatinine at discharge, mmol/l	94 ± 41	93 ± 54	93 ± 40
	• Urea at entry, g/l	7.0 ± 4.4	7.0 ± 3.6	7 ± 3.5
	• Urea at discharge, g/l	8.0 ± 4.2	6 ± 3.6	7 ± 4.4
o KDOQI stage at entry	o I	9 (7.2)	27 (21.6)	36 (14.5)
	o II	66 (53.2)	57 (45.9)	123 (49.5)
	o III	42 (33.8)	37 (29.8)	79 (63.7)
	o IV	5 (4)	3 (2.4)	8 (3.2)
	o V	2 (1.6)	0 (0)	2 (0.8)
o KDOQI stage at discharge	o I	9 (7.2)	10 (8)	19 (7.6)
	o II	53 (42.7)	20 (16)	73 (29.4)
	o III	37 (29.8)	11 (8.8)	48 (19.3)
	o IV	5 (4)	2 (1.6)	7 (2.8)
	o V	2 (1.6)	1 (0.8)	3 (1.2)
	o Not available	18 (14.4)	80 (64.5)	98 (39.5)
CRP, mg/dl		5 ± 19.5	4 ± 16.5	5 ± 18
• Obstructive sleep apnea	• Yes, n (%)	13 (10.4)	23 (18.5)	36 (14.5)
	• AHI mean (/ hour)	21 ± 28	39 ± 25	32 ± 20
	• CPAP rigging, n (%)	8 (6.4)	19 (15.3)	27 (21.7)*
• Neoplasia	• Yes, n (%)	24 (19.3)	12 (9.6)	36 (14.5)*
o Type of neoplasia	o Mammary	7 (5.6)	3 (2.4)	10 (4)
	o Endometrium	0 (0)	1 (0.8)	1 (0.4)
	o Cervix	1 (0.8)	1 (0.8)	2 (0.8)
	o Prostate	7 (5.6)	1 (0.8)	8 (3.2)
	o Renal	2 (1.6)	0 (0)	2 (0.8)
	o Urinary bladder	1 (0.8)	0 (0)	1 (0.4)
	o ENT	1 (0.8)	1 (0.8)	2 (0.8)
	o Broncho-pulmonary	3 (2.4)	0 (0)	3 (1.2)
	o Gastric	0 (0)	1 (0.8)	1 (0.4)
	o Hepatic	1 (0.8)	0 (0)	1 (0.4)
	o Pancreas	0 (0)	1 (0.8)	1 (0.4)
	o Thyroid	1 (0.8)	0 (0)	1 (0.4)
	o Hypophysis	0 (0)	1 (0.8)	1 (0.4)
	o Hematologic	0 (0)	2 (1.6)	2 (0.8)

Notes: *p < 0.05; **p < 0.01.

### Characteristics of AIS among patients in the case group

There was no statistically significant difference between the localization of the stroke concerning the left or right cerebral hemisphere (p = 0.44). A revascularization procedure was performed in 60% of cases. The main modalities were intravenous thrombolysis (41 patients) or intravenous thrombolysis combined with endovascular thrombectomy (21 patients). The median NIH stroke score (NIHSS) was 7 ± 9.5 ( Table 2 ).

**Table 2:** Characteristics of AIS among patients in the case group

Characteristic		AIS Group
**Type of stroke: ischemic**		124 (100%)
**AIS vascular territory**	• Right carotid artery	46 (34.6%)
	• Left carotid artery	63 (47.4%)
	• Right vertebral artery	6 (4.5%)
	• Left vertebral artery	18 (13.5%)
	**(> 1 territory)**	9 (7.2%)
**Revascularization procedure yes: n (%)**		74 (59.6)
**Type of revascularization**	Thrombolysis	41 (33)
	• Thrombectomy	10 (8)
	• Combined (thrombolysis + thrombectomy)	21 (16.9)
	• Craniectomy	1 (0.8)
	• Thrombectomy + angioplasty	1 (0.8)
**NIHSS**		7 ± 9.5

Abbreviations: AIS, Acute Ischemic Stroke; NIHSS, National Institutes of Health Stroke Scale.

### Relation between the risk factors and AIS

In the univariate analysis, risk factors for AIS occurrence were: weight (OR = 0.97; 95% CI [0.96–0.99]; p < 0.001), BMI (OR = 0.95; 95% CI [0.91–1.00]; p = 0.055), age (OR =1.07; 95% CI [1.04–1.10]; p < 0.001), the CHA _2_ DS _2_ -VASc score (OR = 1.44; 95% CI [1.20–1.73]; p < 0.001), the HAS-BLED score (OR = 1.69; 95% CI [1.26–2.26]; p < 0.001), the LVEF (OR = 0.98; 95% CI [0.97–0.99]; p = 0.002), the GFR (OR = 0.98; 95% CI [0.97–0.99]; p = 0.02), high blood pressure (OR =3.23; 95% CI [1.80–5.80]; p < 0.001), the permanent type of AF (OR = 2.52; 95% CI [1.28–4.95]; p = 0.006) and the absence of an anticoagulant treatment (OR = 0.02; 95% CI [0.007–0.05]; p < 0.001).

In the multivariate analysis, the independent risk factors for AIS occurrence were: the CHA _2_ DS _2_ -VASc score (OR = 1.75; 95% CI [1.13–2.70]; p = 0.032), the absence of an anticoagulant treatment OR = 0.19 ; 95% CI [ 0.07–0.51]; p < 0.001 for anticoagulant treatment) and the permanent type of AF OR = 6.31; 95% CI [2.46–16.19]; p < 0.001).

**Figure 1:**
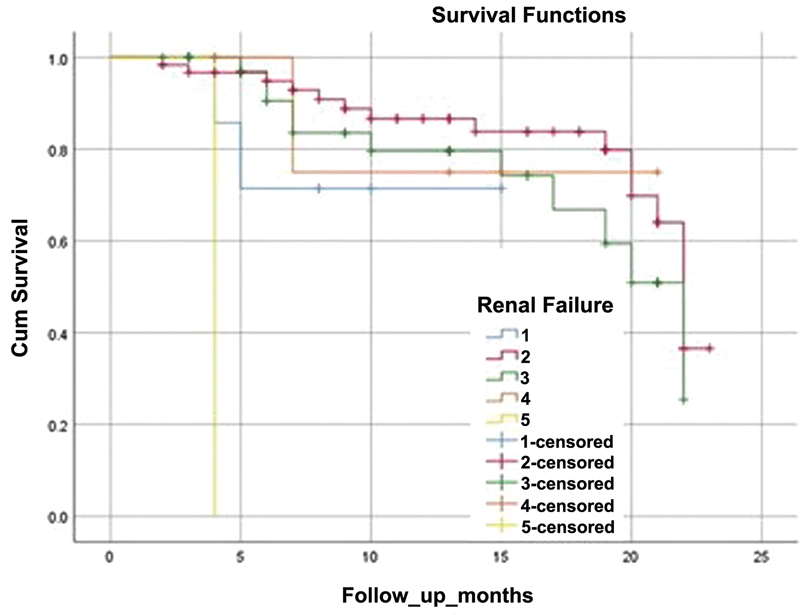
Kaplan-Meyer curve showing the relation between the stage of renal insufficiency and global survival of patients in the AIS group (n = 127) displayed over 23 -months. The baseline was the date of AIS. Surviving patients were censored at the date of data collection.

To assess the impact of the different factors on global survival, Kaplan-Meyer curve was drawn ( Figure 1 ). Chronic kidney disease and COPD were significantly associated with mortality. After a mean 13 months follow-up, the survival of stage 5 KDOQI patients was 0% vs 80% for stage 4 KDOQI patients, 72.5% for stage 3, 75.4 for stage 2 and 71.4% for stage 1. The difference was statistically significant (Log-Rank test, χ ^2^ (1; N = 114) = 27.6 p <0.001).

**Figure 2:**
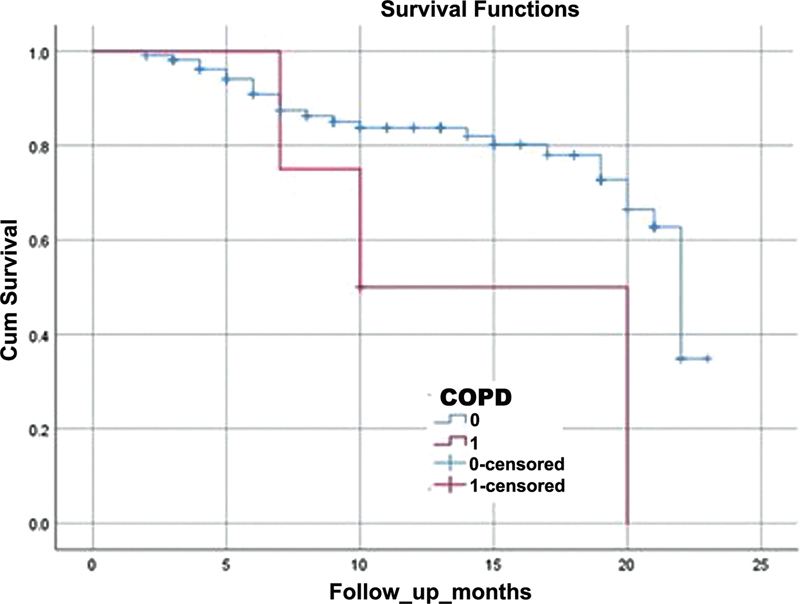
Kaplan-Meyer curve showing the relation between COPD and global survival of patients in the AIS group (n = 127) displayed over 23 -months. The baseline was the date of AIS. Surviving patients were censored at the date of data collection.

The other prognostic factor for global mortality was COPD (Log-Rank test; χ ^2^ (1, N = 114) = 4.45; p =0.035) ( Figure 2 ).

After adjusting for age, sex, CHA2DS2-VASc score and the use of an anticoagulant treatment, we found a statistically significant difference between both groups for weight (79 ± 20 kg vs 84 ± 23 kg; p = 0.036), and a borderline significant difference for CRP (6 ± 14 vs. 3 ± 1; z = 1.87373 ; p = 0.061). With regards to the type of AF, there were more cases of permanent AF in AIS group (20 patients vs 5 patients; p < 0.01) and less paroxysmal (non-significant) and persistent AF (χ ^2^ (1, N = 9) = 7.12; p = 0.007). There were statistically significant correlations between AIS and the permanent type of AF (r = 0.447; p < 0.001) and a borderline correlation between AIS and CRP (r = 0.326; p = 0.056). The ROC curve analysis showed that a CRP threshold value of 3.5 mg/dL at admission had a sensibility of 61.5%, a specificity of 78%, a positive predictive value of 73.5% and a negative predictive value of 66.9% for the occurrence of AIS, but this result did not reach statistical significance (global AUC = 0.71; p = 0.06).

At univariate analysis, the only risk factor predictive for AIS was the permanent type of AF, OR: 8.0 [95% CI 2.5–25.5]; p < 0.001).

## DISCUSSION

The present study showed, on a non-selected population, that the main predictive risk factors for the occurrence of cardio-embolic AIS among patients with NV-AF were the CHA _2_ DS _2_ -VASc score; the absence of an anticoagulant treatment and the permanent type of AF.

Regarding the role of the type of AF, current data shows mixed results. Previous studies have highlighted the clinical interest of including the type of AF in the CHA _2_ DS _2_ -VASc score, for patients with non-paroxysmal AF (persistent or permanent) tend to be at greater embolic risk. 18 Furthermore, in 2019, a study showed that, among patients with non-anticoagulated AF, paroxysmal AF patients had a weaker risk of AIS. 22 Nonetheless, this observation has not been confirmed by other studies, as the type of AF didn't show any predictive interest for AIS occurrence among anticoagulated patients. 22 23 24 25 However, the largest study to date, a systematic review and meta-analysis including 53141 subjects (mean age 65 years) from 16 studies concluded that atrial fibrillation burden > 5 min was associated with increased risk of stroke. 26 This is in accordance with the findings of our study.

In a study involving 2415 AF patients in 2019, 44.7% had paroxysmal AF, 29.4% had persistent AF and 25.9% had permanent AF, which is similar to the prevalence of the different types of AF from our study. 27

In the subpopulation matched for age, sex, CHA _2_ DS _2_ -VASc score and the use of an anticoagulant treatment, we observed a borderline statistically significant correlation between AIS and the CRP value. Our study showed a tendency between systemic inflammation and the occurrence of AIS. Previous studies suggested a threshold value of 3.4 mg/dL that would have a predictive value especially for low and intermediate risk levels of the CHA _2_ DS _2_ -VASc score, 28 other works suggested an increased thrombo-embolic risk above 0.5 mg/dL. 29 Our study strengthened the hypothesis of a threshold value of 3.5 mg/dL.

Despite all these investigations, the risk of AIS in case of an inflammatory syndrome remains controversial. Studies showed non-significant tendencies among patients with AF 30 even though sepsis can trigger AF. 31 In 2006, a meta-analysis highlighted a correlation between increased baseline CRP levels and the occurrence of AIS outside a context of AF. 32 A causal relation is therefore discussed in the literature. In our study, with statistical power considerations aside, the magnitude of the association does not suggest any clinical relevant association. Future studies taking genetics into account might overcome these disparities. 33

In this study, renal failure could not predict the occurrence of AIS among patients with AF. This result echoes other studies. 25 The literature has demonstrated that renal insufficiency increases the risk of AIS 21 34 35 in patients with AF. Such was the case among patients with end-stage renal disease undergoing hemodialysis, where AF was associated with an increased risk for AIS. 36 Other studies even suggested the inclusion of chronic kidney disease in AIS risk stratification scores in AF. 37 38 Further studies including more patients and comparing urea, creatinine and glomerular filtration rate are necessary.

The prevalence of AF at the time of cancer diagnosis is 2.4% and it is estimated that 1.8% of patients will develop AF after diagnosis. 39 The literature indicates that an active cancer should be considered for AIS prevention, but only at the time of diagnosis. 39 It is also admitted that the frequent occurrence of AF in a neoplastic context is due to an adaptive physiologic phenomenon like autonomic nervous system modifications secondary to stress, pain and chronic inflammation. 40 So far, there is no solid proof that an active cancer is a risk factor for AIS among patients with AF. Nonetheless, the causal relation between active cancer and AF is clearly established.

In our study, obstructive sleep apnea (OSA) was not identified as a risk factor for AIS in patients with NV-AF. The prevalence of OSA was 14.5% in our study versus 25% in literature. Several studies showed an association between OSA and AIS or between, AF, OSA and AIS. The presence of OSA would be more predictive for AIS than the CHADS _2_ score. The population of our study was not comparable to these studies regarding OSA prevalence. A lack of statistical power might account for this discrepancy.

Regarding active smoking, after adjusting for age, sex and CHADS-VASc score (but not the use of anticoagulant treatment), our study suggested a correlation with AIS, which was not the case of past smoking. Few studies evaluated the role of past smoking in the occurrence of AIS. But they concluded unequivocally that active smoking, and to lesser extent past smoking, favors the onset of AF through physiologic modifications. Smoking is hence a modifiable risk factor of AF. It is also independently associated with AIS. With 7.5 million deaths worldwide in 2015, further studies would be necessary to assess the inclusion of active smoking into the CHA _2_ DS _2_ -VASc score.

In this study, COPD was associated with increased mortality in AIS group. The literature is poor on this topic, but a Chinese epidemiologic study over 27 years identified COPD as one of the most frequent cause of global mortality. Its prevalence among AF patients is estimated between 10% and 15%. A higher thrombo-embolic risk during COPD exacerbations is discussed independently of AF. Hospitalizations for COPD exacerbations have a worse prognosis with a lower survival. This specific fragility might be explained in case of AIS by the frequent aspiration pneumonia after endovascular thrombectomy or secondary to swallowing dysfunction.

End stage renal disease (stage 5 of KDOQI classification) was also associated with higher mortality among patients hospitalized for cardio-embolic AIS. The prevalence of AF among these patients is 11.6%. Among those, the incidence of AIS 5.2% per year and the global mortality is 26.9% per year. On the contrary, the incidence of AIS among end stage renal disease patients without AF is 1.9% per year and the global mortality is 13.4% per year, which echoes our results ( Figure 1 ).

We found no significant difference in the localization of the ischemic stroke (left vs. right hemisphere). This is in accordance with current data from the literature. 41

### Study limitations

The study has statistically significant differences in patients' characteristics between both groups, which impaired their comparability: age, CHA _2_ DS _2_ -VASc score or the use of anticoagulant treatment.

By adjusting for for age, sex, CHA _2_ DS _2_ -VASc score and the use of anticoagulant treatments, we corrected as much as possible the previous bias by case-control matching. However, the small sample size decreased the statistical power, which limits the extent of our conclusions.

This is a retrospective observational study. It suffers of all the limitations of such studies, especially regarding patients recruitment and confusion factors. The longitudinal follow-up in the survival analysis varied between the patients as a result of the censoring at the date of data collection.

Also, including patients from 2 specific departments of the same hospital might have introduced a selection bias.

The main axes highlighted in our study arouse the need for larger studies for validation.

Also, in the absence of continuous ECG monitoring before and during the occurrence of the strokes, it is possible that short episodes of paroxysmal atrial fibrillation went undetected. This may change the results of this study.

In conclusion, the CHA _2_ DS _2_ -VASc score, the absence of anticoagulants and the permanent type of AF were the main predictive factors of cardio-embolic AF. Other than these three factors, active smoking and systemic inflammation were potential risk factors for AIS among patients with NV-AF. Further and larger studies are necessary to draw firm conclusions on this topic.
